# Effects of Schiff Base Formation and Aldol Condensation on the Determination of Aldehydes in Rice Wine Using GC-MS

**DOI:** 10.3390/molecules22040618

**Published:** 2017-04-11

**Authors:** Ji Hye Han, Sang Mi Lee, Young-Suk Kim

**Affiliations:** Department of Food Science and Engineering, Ewha Womans University, Seoul 120-750, Korea; wlgo9698@naver.com (J.H.H.); smlee78@ewha.ac.kr (S.M.L.)

**Keywords:** alcoholic beverage, aldehydes, pH, amino acids, Schiff base formation, aldol condensation

## Abstract

The Schiff base reaction and aldol condensation that occur during sample preparation can lead to the reduction of aldehyde content in the analysis of traditional Korean rice wine, *makgeolli*. The contents of aldehydes were decreased, whereas those of hydroxy carbonyl compounds were increased by increasing the pH. In the presence of added amino acids, the levels of aldehydes in *makgeolli* were reduced as the amount of the amino acid alanine increased. Also, the contents of hydroxyl carbonyl compounds were reduced by alanine addition as compared to the control. Therefore, the determination of aldehydes can be affected by pH and the amount of amino acids, which can vary during fermentation and storage of alcoholic beverages because pH and amino acids affect Schiff base formation and aldol condensation.

## 1. Introduction

Interest in the analysis of carbonyl compounds in food items and drinks has increased significantly in recent years because some of these compounds are responsible for the unpleasant odors and potential adverse health effects of alcoholic beverages [1,2,3,4]. In alcoholic beverages, carbonyl compounds, especially aldehydes, which are formed from by-products of alcoholic fermentation and alcohol oxidation, can affect the quality of beverages because they exhibit unique odor notes, in some instances even at low levels [1,5]. Also, these carbonyl compounds can be produced from major macromolecules such as proteins, lipids and carbohydrates from the raw materials used [1]. Therefore, the formation of carbonyls is in part responsible for the quality of alcoholic beverages as well as their safety.

The International Agency on Research for Cancer (IARC) has classified acetaldehyde, which is the major aldehyde and generally comprises more than 90% of the total aldehydes in wines and distilled alcoholic beverages [6], into group 2B, and acetaldehyde consumed with alcoholic beverages was classified as group 1 with respect to the occurrence of cancer in humans (IARC, 2015).

Aldehydes are present at various levels in alcoholic beverages [7]. The highest aldehyde level normally occurs during fermentation, when the yeast is in the most vigorous phase, although the amount of total aldehydes seems to depend on the types of yeasts and the composition of nutrients used [4]. Large amounts of aldehydes in wine can result from a deficiency of nutrient substances during the fermentation of grapes, which leads to the delayed formation of ethyl alcohol [8]. Diverse reactions such as the oxidation of alcohols, autoxidation of fatty acids, and Strecker degradation of amino acids can also produce aldehydes in alcoholic beverages.

Carbonyl compounds can be determined by several methods. Enzymatic assay can be used for the determination of acetaldehyde, according to the official methods of the National Research Institute of Brewing (NRIB) and the American Society of Brewing Chemistry (ASBC, 2013). Colorimetric methods for determining acetaldehyde are recommended by the Ministry of Food and Drug Safety (MFDS) and the Food Safety and Standards Authority of India (Fassai). The main advantage of both enzymatic assays and colorimetric methods is that there is no need to use additional instruments, although these methods are less selective and sensitive than instrumental analyses such as gas chromatography (GC) and high performance liquid chromatography (HPLC) [9].

HPLC and GC are most frequently applied to the analysis of carbonyl compounds due to their high resolution and accuracy [1]. Aldehydes and other volatile compounds in alcoholic beverages have been analyzed using gas chromatography-flame ionization detector (GC-FID) combined with direct injection according to the official method of the International Organisation of Vine and Wine (OIV) and the European Union (EU) (EC No. 2870/2000). In general, gas chromatography-mass spectrometry (GC-MS) combined with solid-phase micro-extraction (SPME) has been recommended for the analysis of low molecular mass aldehydes in alcoholic beverages [1]. Because aldehydes are highly volatile and reactive, they usually require derivatization to achieve adequate recovery and sensitivity [10]. SPME has been also applied to determine aldehydes after derivatization in beer [11], wine and spirits [1].

However, determination of carbonyl compounds in alcoholic beverages is limited by problems such as low precision and recovery, mainly because these compounds exhibit high reactivity and volatility [10]. The main reactions related to the loss of aldehydes are due to the formation of Schiff bases and aldol condensation. The formation of Schiff bases usually occurs by the reaction of an aldehyde or ketone with a primary amine under acid or base catalysis or with heat [12], whereas aldol condensation is the reaction of carbonyl compounds, such as nucleophiles, with electrophiles such as aldehydes or ketones to form β-hydroxyl carbonyl compounds [13]. Although Schiff base formation and aldol condensation can be expected for aldehydes, leading to the loss of those compounds during analysis, there have been no studies to verify their effects on the determination of carbonyl compounds in alcoholic beverages. These reactions are affected by pH and by other compounds such as amino acids in samples, so investigations to determine aldehydes that take into consideration changes in sample conditions are needed. In this study, the effects of Schiff base formation and aldol condensation on the determination of aldehydes in *makgeolli*, a traditional rice wine in Korea, were investigated by comparing the amounts and types of aldehydes and hydroxyl carbonyl compounds formed at different pH values and amino acid levels. *Makgeolli* was chosen because its contents of amino acids and peptides as well as its pH can be varied depending on manufacturing processes and storage conditions, which are closely related to its quality.

## 2. Results and Discussion

### 2.1. Effect of pH on the Determination of Aldehydes in Makgeolli

*Makgeolli* contains about 7% ethanol, which is lower than that of either wines or spirits. Also, *makgeolli* has about 60%~70% of proteins in the dry base which originates from the raw materials [14]. It has been suggested that proteins are readily degraded into low molecular weight peptides and amino acids during fermentation [14]. Aldehydes can also be produced by yeast fermentation or ethanol oxidation. Lee et al. showed that there are 10 aldehydes in *makgeolli* fermented from Rhizopus japonicas, and that aldehydes make up the main volatile compounds.

The adjustment of pH values of samples was performed to determine the effect of aldol condensation and Schiff base reaction on the reduction of aldehydes in *makgeolli*. The contents of aldehydes, except for acetaldehyde, were significantly decreased whereas those of hydroxy carbonyl compounds, except for hydroxyl acetaldehyde, were increased at alkaline pH (Table 1). These results could be explained by aldol condensation.

Hydroxyl carbonyl compounds are intermediates of aldol condensation and are more readily produced in basic states. Hydroxyl carbonyl compounds can be converted to unsaturated carbonyl enones, which are stable and irreversible [15]. Most hydroxyl carbonyl compounds are increased at basic pH, whereas hydroxyl acetaldehydes are increased by the neutral state and are decreased under basic conditions. This phenomenon can occur due to the conversion of a hydroxyl acetaldehyde to an unsaturated carbonyl enone, which is relatively stable [15]. However, the reduction of aldehydes also could be explained by Schiff base formation, because it is accelerated by base catalysis.

It has been demonstrated that proline and its derivatives can act as catalysts. The amine group is known to activate the aldol donor molecules by converting them into enamines, while the carboxylic acid provides a hydrogen bond to the acceptor. The enamine functionality is derived by the reaction of a carbonyl compound with a secondary amine [16]. The enamine attacks the carbonyl groups of aldehydes, which leads to aldol condensation [17]. Therefore, a secondary amine can catalyze aldol condensation.

**Figure 1:**
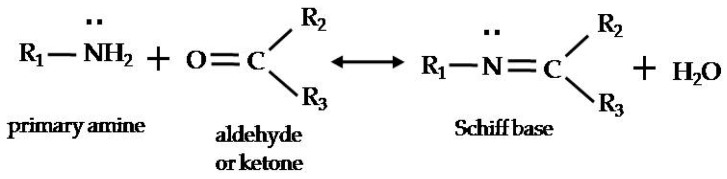
General scheme for formation of Schiff base [18].

The Schiff base is formed by the reversible reaction between a primary amine and a carbonyl compound (Figure 1) [18]. If both the primary amine and the carbonyl compound are present, a secondary amine is produced by the Schiff base reaction. Then, an enamine is formed by reaction of the secondary amines with other carbonyl compounds. Thus, it can be regarded that a secondary amine from Schiff base formation activates aldol condensation in *makgeolli*. To investigate the effect of secondary amine formation on aldol condensation, a secondary amine, proline, was added to samples. Table 2 indicates that the contents of hydroxy carbonyl compounds, except for 3-hydroxy-2-butanone, showed a decreasing tendency in accord with the increasing amounts of proline, although some of them were not statistically different among samples. This outcome could result from the activation of aldol condensation by the presence of a secondary amine and by the conversion of hydroxyl carbonyl compounds into unsaturated carbonyl enones, which is an irreversible reaction.

The contents of organic acids generally increased during the fermentation period [15]. On the other hand, the maximum content of citric acid, which represents the largest amount of carboxylic acid in *makgeolli*, was obtained at the seventh day of fermentation, after which its levels decreased [15]. The change in the amounts of organic acids can vary based on variations in pH during the fermentation process in the preparation of *makgeolli*, which can ultimately affect the determination of aldehydes.

### 2.2. Effects of Amino Acids on the Determination of Aldehydes in Makgeolli

It is known that free amino acids and peptides are produced from proteins, which are the major raw material in rice, during fermentation of *makgeolli* [19]. It was reported that *makgeolli* contains free amino acids that contribute to the sweet, savory, sour, and bitter tastes [20]. Kang et al. reported that commercial *makgeolli* is composed of 0.2%~1.27% of reducing sugars, 0.47%~0.95% of amino acids, and 0.28%~0.57% of total acids. Alanine, phenylalanine, tyrosine, leucine, glutamic acid, and proline were identified as the major amino acids in *makgeolli* [15].

Therefore, the effects of amino acids with amine groups on the determination of aldehydes were studied in a subsequent experiment, investigated separately from the pH effect. In order to investigate the effect of Schiff base formation and the quantitative effect of amino acids, aliquots of 50 mg/L and 100 mg/L of alanine were added to samples of *makgeolli*. Alanine has been identified as a major amino acid in *makgeolli* and its levels increased in accordance with different parts of the fermentation [11]. The contents of all aldehydes were reduced as the amount of added alanine increased (Table 3). In particular, the contents of acetaldehyde, 2-methylpropanal, 2-methylbutanal, and 3-methylbutanal were significantly decreased as the addition of alanine increased. However, 5-methyl-2-furancarboxaldehyde, 2-methylbutanal, 2-methylpropanal, and benzeneacetaldehyde were slightly decreased, as compared to the controls without the addition of alanine. The contents of some aldehydes were significantly changed by the addition of amino acids, and were also highly affected by the level of alanine. Therefore, amino acids present at different levels during the fermentation of *makgeolli* [15] can lead to variations in the contents of aldehydes.

## 3. Materials and Methods

### 3.1. Materials

Ethanol (>99.9%) was purchased from Sigma-Aldrich (Sigma-Aldrich, St. Louis, MO, USA) for standards preparation. 4-Methyl-1-pentanol, an internal standard (ISTD) compound, was also obtained from Sigma-Aldrich. dl-alanine and dl-proline were purchased from Sigma-Aldrich and Oasis MAX cartridge 6 cc with stationary phase was purchased from Waters (Waters, Milford, MA, USA).

### 3.2. Sample Preparation

Commercial rice wine (*makgeolli*) samples were purchased from a local market in Seoul, Republic of Korea. All the samples used were manufactured from the same lot on the same day. All the samples were mixed thoroughly, divided into about 250 mL, and stored at −70 °C. Then, they were thawed at 1 °C overnight before conducting the experiments.

#### 3.2.1. pH

4.9 mL of *makgeolli* was transferred into a 50-mL falcon tube (Becton Dickinson, Cowley, Oxford, UK). 100 μL of 4-methyl-1-pentanol, an internal standard (50 mg/L in ethanol), was added into the sample before the sample was adjusted to neutral or basic condition using 1 M sodium hydroxide (NaOH). The sample was mixed using a vortexer and centrifuged for 10 min at 4 °C and 3500 rpm. Then supernatant was transferred to a test tube and vortexed. Finally, 1 mL of supernatant was placed in a 2-mL amber vial.

#### 3.2.2. Addition of Amino Acid

4.4 mL of *makgeolli* was transferred into a 50-mL falcon tube. 100 μL of 4-methyl-1-pentanol, an internal standard (50 mg/L in ethanol), and 0.5 mL of alanine (50 mg/L or 100 mg/L in water) were added into the sample. The sample was mixed using vortexer and centrifuged for 10 min at 4 °C and 3500 rpm. Then supernatant was transferred to a test tube and vortexed. Finally, 1 mL of supernatant was placed in a 2-mL amber vial.

#### 3.2.3. Addition of Secondary Amine 

4.7 mL of *makgeolli* was transferred into a 50-mL falcon tube. 100 μL of 4-methyl-1-pentanol, an internal standard (100 mg/L in ethanol), and 0.1 mL of proline (50 mg/L and 100 mg/L in water) were added into the sample. The sample was mixed using a vortexer and centrifuged for 10 min at 4 °C, and 3500 rpm. Then supernatant was transferred to a test tube and vortexed. Finally, 1 mL of supernatant was placed in a 2-mL amber vial.

### 3.3. Gas Chromatography-Mass Spectrometry (GC-MS) Analysis

GC-MS analysis was performed using a HP 7890B gas chromatograph (Agilent Technologies, Santa Clara, CA, USA) coupled with a 5977A mass selective detector (MSD) (Agilent Technologies) and a multi-purpose sampler MPS 2 (Gerstel, Mülheim an der Ruhr, Germany). HP-innowax fused silica capillary column (30 m length × 0.25 mm internal diameter × 0.25 μm film thickness; Agilent Technologies) was used with helium, a carrier gas, at a constant flow rate of 1 mL/min. Injection volume was 1.0 μL with split mode (20:1). The oven temperature program started at 40 °C initially and was held for 3 min, then was raised to 200 °C at 5 °C/min and held at 200 °C for 3 min. Post run was held at 180 °C for 2 min. Inlet, detector transfer line and mass source temperatures were 250, 280, 230 °C, respectively. MS was operated in the electron impact (EI) ion source mode at 70 eV with a scan range of 25~350 a.m.u. Ethanol (>99.9%) was purchased from Sigma-Aldrich (Sigma-Aldrich, St. Louis, MO, USA) for standard preparation. 4-Methyl-1-pentanol, an internal standard (ISTD) compound, was also obtained from Sigma-Aldrich. dl-alanine and dl-proline were purchased from Sigma-Aldrich and Oasis MAX cartridge 6 cc with stationary phase was purchased from Waters (Waters, Milford, MA, USA).

### 3.4. Statistical Analyses

Analysis of variance (ANOVA) was performed with a general linear model procedure in SPSS (version 12.0, Chicago, IL, USA) to evaluate significant differences of compounds in samples. Post-hoc analysis was determined using Duncan’s multiple comparison test (*p* < 0.1).

## 4. Conclusions

The determination of aldehydes is affected by Schiff base formation and by aldol condensation, reactions which are sensitive to pH and the presence of amino acids. Therefore, during the determination of aldehydes, the conditions of the samples should be taken into account because pH and the amounts of amino acids can vary during fermentation and storage of alcoholic beverages or foods and can affect the results of the aldehyde measurements.

## Figures and Tables

**Table 1:** Comparison of the contents of aldehydes and hydroxyl carbonyl compounds at different pH values.

Compounds	Relative Peak Area (Mean ± SD) ^1^		
	pH 4	pH 7	pH 11
acetaldehyde	2.198 ± 0.641 a ^2^	1.919 ± 0.467 a	1.802 ± 0.197 a
2-methylpropanal	1.755 ± 0.440 a	1.482 ± 0.203 a	0.755 ± 0.131 b
2-methylbutanal	1.065 ± 0.351 a	1.053 ± 0.270 a	0.271 ± 0.053 b
3-methylbutanal	3.877 ± 1.151 a	3.051 ± 0.580 a	1.207 ± 0.092 b
benzeneacetaldehyde	0.505 ± 0.182 a	0.400 ± 0.119 a	0.126 ± 0.030 b
methional	0.159 ± 0.042 a	0.146 ± 0.027 a	ND b
5-methyl-2-furancarboxaldehyde	0.278 ± 0.105 a	ND ^3^ b	ND b
3-hydroxy-2-butanone	0.136 ± 0.033 c	0.295 ± 0.093 b	0.614 ± 0.143 a
1-hydroxy-2-propanone	2.709 ± 0.817 b	6.388 ± 1.941 b	13.212 ± 4.121 a
hydroxy acetaldehyde	0.981 ± 0.504 b	1.937 ± 0.347 a	1.605 ± 0.094 a
1-hydroxy-2-butanone	0.146 ± 0.054 b	0.280 ± 0.109 b	0.634 ± 0.265 a
2-hydroxy-2-cyclopenten-1-one	0.702 ± 0.028 b	0.914 ± 0.055 b	1.331 ± 0.105 a

^1^ Volatile compounds were calculated with the relative peak ratio of their peak areas to that of internal standard (*n* = 3) ± standard deviation; ^2^ Different letters indicate significant differences (*p* < 0.1) between three different samples according to three different pH values by Duncan’s multiple range test; ^3^ ND = not detected.

**Table 2:** Comparison of the contents of hydroxyl carbonyl compounds by increasing proline.

Compounds	Relative Peak Area (Mean ± SD) ^1^		
	Control (*makgeolli*)	Control +50 ppm Proline	Control +100 ppm Proline
3-hydroxy-2-butanone	0.037 ± 0.034 b ^2^	0.075 ± 0.017 a	0.040 ± 0.010 a,b
1-hydroxy-2-propanone	0.116 ± 0.045 a	0.088 ± 0.019 a,b	0.048 ± 0.014 b
hydroxy acetaldehyde	0.135 ± 0.124 a	0.028 ± 0.018 a	0.028 ± 0.005 a
2-hydroxy-2-cyclopenten-1-one	0.177 ± 0.153 a	0.102 ± 0.053 a	0.042 ± 0.001 a

^1^ Volatile compounds were calculated with the relative peak ratio of their peak areas to that of internal standard (*n* = 3) ± standard deviation; ^2^ Different letters indicate significant differences (*p* < 0.1) between three different samples according to three different proline contents by Duncan’s multiple range test.

**Table 3:** Comparison of the contents of aldehydes by adding alanine.

Compounds	Relative Peak Area (Mean ± SD) ^1^		
	Control (*makgeolli*)	Control +50 ppm Alanine	Control +100 ppm Alanine
acetaldehyde	2.365 ± 0.239 a ^2^	1.726 ± 0.343 b	1.335 ± 0.147 b
2-methylpropanal	2.111 ± 0.175 a	1.766 ± 0.505 a	1.186 ± 0.097 b
2-methylbutanal	1.427 ± 0.218 a	1.021 ± 0.419 a,b	0.604 ± 0.204 b
3-methylbutanal	3.599 ± 0.406 a	3.519 ± 0.773 a	2.403 ± 0.140 b
benzeneacetaldehyde	0.599 ± 0.135 a	0.444 ± 0.111 a,b	0.407 ± 0.095 b
methional	0.175 ± 0.025 a	0.169 ± 0.053 a	0.128 ± 0.026 a
5-methyl-2-furancarboxaldehyde	0.289 ± 0.102 a	0.262 ± 0.188 a	0.088 ± 0.011 a

^1^ Volatile compounds were calculated with the relative peak ratio of their peak areas to that of internal standard (*n* = 3) ± standard deviation; ^2^ Different letters indicate significant differences (*p* < 0.1) between three different samples according to three different alanine contents by Duncan’s multiple range test.
